# Multichannel anodal tDCS over the left dorsolateral prefrontal cortex in a paediatric population

**DOI:** 10.1038/s41598-021-00933-z

**Published:** 2021-11-02

**Authors:** Maike Splittgerber, Christoph Borzikowsky, Ricardo Salvador, Oula Puonti, Kiriaki Papadimitriou, Christoph Merschformann, Maria Chiara Biagi, Tristan Stenner, Hannah Brauer, Carolin Breitling-Ziegler, Alexander Prehn-Kristensen, Kerstin Krauel, Giulio Ruffini, Anya Pedersen, Frauke Nees, Axel Thielscher, Astrid Dempfle, Michael Siniatchkin, Vera Moliadze

**Affiliations:** 1 Institute of Medical Psychology and Medical Sociology, University Medical Center Schleswig Holstein, Kiel University, Kiel, Germany; 2 Institute of Medical Informatics and Statistics, University Hospital Schleswig Holstein, Kiel University, Kiel, Germany; 3 Neuroelectrics, Barcelona, Spain; 4 Danish Research Centre for Magnetic Resonance, Centre for Functional and Diagnostic Imaging and Research, Copenhagen University Hospital Hvidovre, Hvidovre, Denmark; 5 Department of Child and Adolescent Psychiatry, Center for Integrative Psychiatry Kiel, University Medical Center Schleswig-Holstein, Kiel, Germany; 6 Department of Child and Adolescent Psychiatry and Psychotherapy, University of Magdeburg, Magdeburg, Germany; 7 Department of Psychology, Faculty of Human Sciences, MSH Medical School Hamburg - University of Applied Sciences and Medical University, Hamburg, Germany; 8 Center for Behavioral Brain Sciences, Magdeburg, Germany; 9 Clinical Psychology and Psychotherapy, Department of Psychology, University of Kiel, Kiel, Germany; 10 Clinic for Child and Adolescent Psychiatry and Psychotherapy, Protestant Hospital Bethel, University of Bielefeld, Campus Bielefeld Bethel, Bielefeld, Germany

**Keywords:** Neuroscience, Cognitive neuroscience

## Abstract

Methodological studies investigating transcranial direct current stimulation (tDCS) over the left dorsolateral prefrontal cortex (lDLPFC) in paediatric populations are limited. Therefore, we investigated in a paediatric population whether stimulation success of multichannel tDCS over the lDLPFC depends on concurrent task performance and individual head anatomy. In a randomised, sham-controlled, double-blind crossover study 22 healthy participants (10–17 years) received 2 mA multichannel anodal tDCS (atDCS) over the lDLPFC with and without a 2-back working memory (WM) task. After stimulation, the 2-back task and a Flanker task were performed. Resting state and task-related EEG were recorded. In 16 participants we calculated the individual electric field (E-field) distribution. Performance and neurophysiological activity in the 2-back task were not affected by atDCS. atDCS reduced reaction times in the Flanker task, independent of whether atDCS had been combined with the 2-back task. Flanker task related beta oscillation increased following stimulation without 2-back task performance. atDCS effects were not correlated with the E-field. We found no effect of multichannel atDCS over the lDLPFC on WM in children/adolescents but a transfer effect on interference control. While this effect on behaviour was independent of concurrent task performance, neurophysiological activity might be more sensitive to cognitive activation during stimulation. However, our results are limited by the small sample size, the lack of an active control group and variations in WM performance.

## Introduction

Transcranial direct current stimulation (tDCS) is a promising neuromodulatory technique in research and clinical application^1^ (for review see^2^). Although the use of tDCS in children and adolescents is increasingly being investigated, important insights into how tDCS affects and interacts with the developing brain are missing. Previous studies in the motor cortex show that findings on tDCS effects obtained in adults cannot simply be assumed to be valid for tDCS application in children and adolescents^3,4^. Compared to adults, children show different conductivity of the skull tissue, different white and gray matter content and cerebrospinal fluid (CSF) volume as well as a smaller brain-scalp distance, all of which influence the electric-field (E-field) distribution^5,6^. However, due to differences in cortical architecture, receptor distribution and anatomical factors, it is not clear if findings from motor cortex are transferable to other cortical areas^7^.

The left dorsolateral prefrontal cortex (DLPFC) is an area often used as target region for electrical stimulation, due to its role for various cognitive and executive functions such as working memory (WM) or decision making^8,9^. tDCS was shown to modulate neuronal activity in the left dorsolateral prefrontal cortex (lDLPFC) and associated cognitive functions in adults^10,11^ and in clinical samples in children and adolescents^12,13^. However, to date, no methodological studies have been conducted on the effects of tDCS over the lDLPFC in normally developed children and adolescents.

While tDCS studies show partially no linear dependency between applied current intensity and stimulation effects^14–16^, studies modelling the individual current flow inside the brain indicate that higher target field strengths may lead to stronger stimulation effects^17,18^. Previous studies that stimulated the lDLPFC using tDCS have mostly used classical bipolar montages, which produce a relative diffuse electric field (E-field) and poor targeting^19,20^. An alternative could be optimised multichannel montages, for which modelling studies predict a comparatively strong but also focal stimulation of the target area^21,22^. For adults, an increased effectiveness of an optimised multichannel montage has already been shown for motor cortex stimulation^23^ as well as lDLPFC stimulation^24^.

Studies in adults suggest that cognitive engagement during stimulation has an impact on the type and strength of tDCS after-effects^25,26^. Regarding lDLPFC stimulation, both a superiority of online^26,27^ and of offline stimulation have been proposed^28,29^. Especially in the context of a potential therapeutic use of tDCS, it is relevant to generate broad, transferring effects. According to the flexible hub theory, the general enhancement of the lDLPFC as an after-effect of tDCS might improve different executive functions, based on the current task related demands^30,31^. Unfortunately, online and offline stimulation is rarely compared within the same study.

An important tool to illustrate stimulation effects is provided by neurophysiological correlates such as event-related potentials (ERPs) and event-related and resting state oscillatory power recorded by EEG. Studies in adults demonstrate effects of anodal tDCS (atDCS) over the lDLPFC on neurophysiological activity. atDCS led to increased task related N2 and P3 amplitudes^32,33^. Additionally, theta and alpha oscillatory power have been shown to be influenced by tDCS^24,34^. Also, changes in resting state oscillatory power have been demonstrated following atDCS^33,35^. However, several studies did not prove an effect of tDCS over the lDLPFC on resting state oscillatory power^36–38^.

The current study aimed at investigating the previously mentioned factors using multichannel atDCS over the lDLPFC in children and adolescents (10–17 years). We explored whether atDCS effects are influenced by concurrent task performance during stimulation and individual anatomy. As outcomes we used behavioural and neurophysiological variables. We expected atDCS to lead to improved performance and corresponding EEG correlates in a 2-back WM task (target task). Further, we expected stimulation effects to be influenced by concurrent task performance during stimulation. To investigate atDCS transfer effects, we used a Flanker task (non-target task) that investigates interference control^39^. Following the flexible hub theory, we expected that atDCS also improves Flanker task performance and related neurophysiological activity. Furthermore, we assumed that the effects of atDCS are modulated by individual anatomy, with higher E-field distributions in the target area leading to stronger atDCS effects. Because respective research in typically developing children and adolescents is lacking, we also investigated aspects of tolerability for multichannel atDCS.

## Materials and methods

### Participants

**Figure 1:**
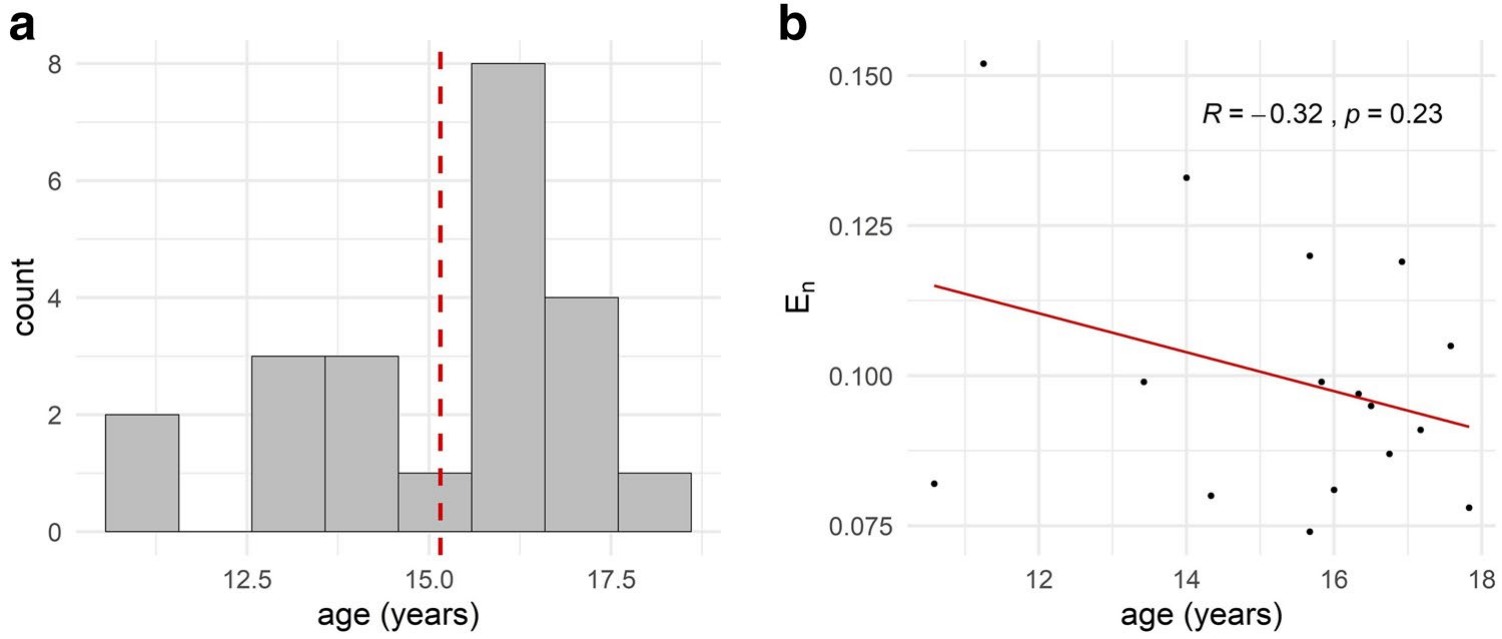
Age and individual normal E-field component (E_*n*_). **(a)** Age distribution of the whole sample size (n = 22). Red line indicates mean age of 15.18 years. **(b)** Correlation of age and normal E-field component (E_*n*_) (n = 16).

The study was approved by the local ethics committee of the Medical Faculty, Kiel University, Kiel, Germany and was carried out in accordance with the latest revision of the Declaration of Helsinki (Clinical trial registration number: German Clinical Trials Register DRKS00008207, 31/08/2017). This study is part of the EU research project STIPED (Stimulation in pediatrics; grant agreement No 731827). All participants and their parents gave written informed consent prior to the experiment. In total, 34 participants were recruited. After a screening procedure we included 29 healthy children and adolescents between 10 and 18 years. Due to study dropout and technical problems during data recording, a total of 22 participants were included in the final data analyses (14 females, mean age: 15.18 years, SD: 1.9, Fig. 1a). Except for one case, the reason given for study termination was that study participation was too time-consuming. One 13-year old girl developed an epileptic disease during her study participation and had to be excluded^40^. Exclusion criteria were an IQ score < 80 (CFT-20-R, Grundintelligenztest Skala 2—Revision)^41^, abnormalities during pregnancy or birth, past or present chronic medical conditions, epileptic seizure(s) in the past or in the family, substance consumption or regular medication, any body electronic devices or implants and pregnancy. Health and social impairments were further assessed using the CBCL (Child Behavior Checklist^42^), FBB-ADHS (Fremdbeurteilungsbogen für Aufmerksamkeitsdefizit-/Hyperaktivitätsstörungen^43^) and SRS (Social Responsiveness Scale^44^). All participants’ characteristics can be found in Table 1. We did not control for handedness since previous studies and metanalyses report no effect of handedness on tDCS effects above the lDLPFC^10–12,45^.

**Table 1:** Participants characteristics.

	Mean ± standard deviation	Exclusion criteria
Sex	14 female, 8 male	
Age	15.12 years ± 1.9	10 < age > 18
CFT-20-R	106.68 ± 8.91	IQ < 80
CBCL Competence	59.86 ± 7.66	T < 37
CBCL Problems	48 ± 8.14	T > 69
FBB-ADHS	0.19 ± 0.18	KW > 0.5
SRS	45 ± 9.39	T > 60

*CFT-20-R* Grundintelligenztest Skala 2-Revision, *CBCL* child behavior checklist, *FBB-ADHS* Fremdbeurteilungsbogen für Aufmerksamkeitsdefizit-/Hyperaktivitätsstörungen, *SRS* social responsiveness scale.

### Experimental design

**Figure 2:**
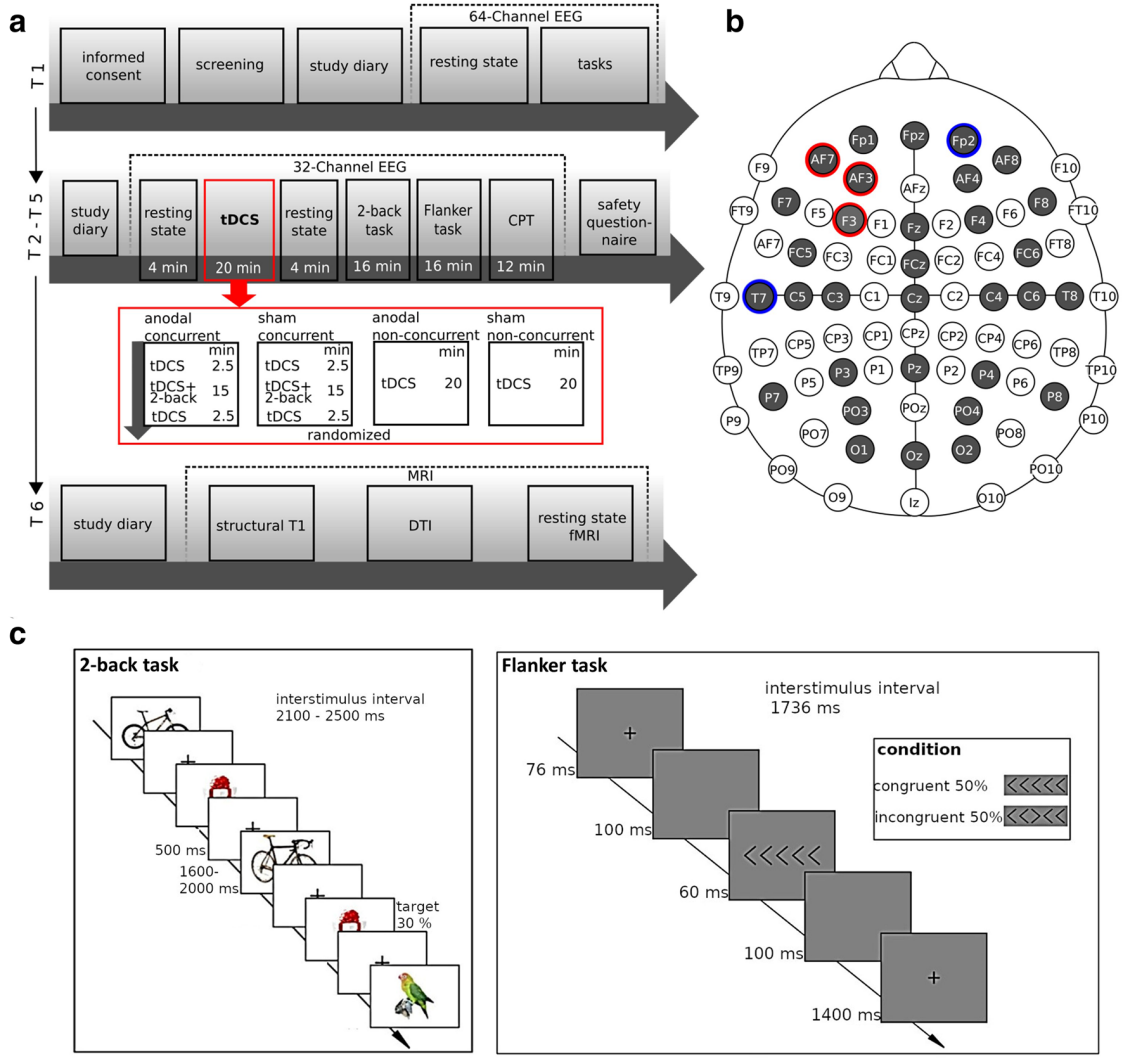
Experimental Design. **(a)** Time-course of the experiment. Each participant was stimulated four times (T2–T5) with the following condition: anodal (atDCS) with 2-back performance, sham tDCS with 2-back performance, atDCS without task performance, sham tDCS without task performance. After stimulation, the participants always performed the 2-back task, Flanker task and Continuous Performance Task (CPT). EEG was recorded at rest (pre and post tDCS), during stimulation and after stimulation during task performance. At an optional session (T6) MRI data for individual modelling was obtained. **(b)** Electrode montage for EEG and multichannel stimulation. Red circles represent anodal, blue circles reference electrodes. Grey colour indicates EEG electrodes. **(c)** Tasks. In the 2-back task participants had to decide whether a currently presented picture was identical to the picture shown 2 steps back. In the Flanker task participants had to indicate via button press if the middle target arrow pointed to the right or to the left. Figures were created using Inkscape (Version 0.92, https://inkscape.org).

We used a randomised, sham-controlled, double-blind, crossover study design (see Fig. 2a). Participants underwent six experimental sessions: one screening and baseline measurement (T1) followed by four stimulation sessions (T2–T5) and one optional MRI session (T6). In the stimulation sessions each participant received four different stimulation conditions in randomised order: atDCS with or without concurrent 2-back task performance and sham tDCS with or without concurrent 2-back task performance. The minimum period between stimulation sessions for a single participant was 7 days. At the start of each stimulation session, participants filled in a diary, asking for any adverse events since the last session and their current mood and motivation. Next, we recorded a resting state EEG (2 min eyes open, 2 min eyes closed), followed by 20 min stimulation. In case of concurrent task performance during stimulation, the 2-back task started after 2.5 min of tDCS and ended 2.5 min before the end of stimulation. During non-concurrent stimulation, participants were instructed to sit relaxed with opened eyes. After stimulation, we recorded a second resting state EEG (2 min eyes open, 2 min eyes closed). Afterwards participants performed the 2-back task, a Flanker task and a Continuous Performance Task (CPT) during EEG recording. Eventually, participants filled in a questionnaire on safety, tolerability and blinding of stimulation. The present study is limited to the analysis of the stimulation sessions (T2–T5). Of the tasks performed, the 2-back and Flanker task were evaluated, while the CPT was performed for later comparisons with patient groups.

### Tasks and stimuli

All tasks were programmed using the software Presentation^®^ (Version 20.0, Neurobehavioral Systems, Inc., Berkeley, CA, https://www.neurobs.com/). In the 2-back participants had to decide whether a currently presented picture was identical to the picture shown two steps back (Fig. 2c). Pictures were taken from the Stark Lab Mnemonic Similarity Task (MST^46^). To make the task more demanding, we included lure pictures. Participants had to press the right (non-target trial) or the left (target trial) mouse button in each trial. The task lasted approximately 16 min and contained 366 trials with 30% target trials. Each trial consisted of 500 ms picture presentation followed by fixation cross presentation jittered between 1600 and 2000 ms duration, resulting in a trial duration of 2100–2500 ms. This 2-back task was previously included in our study with healthy adults^24^ and is based on the verbal WM n-back task^47,48^. At baseline measurement (T1) and at each stimulation visit (T2–T5) participants performed a short training (23 trials) to ensure the task was understood correctly. The training was repeated until participants achieved an accuracy of at least 15 (65%) correctly answered trials.

In the Flanker task (Fig. 2c), stimuli consisted of five arrows. This task is a modified version of the Eriksen Flanker task^39^ and was previously used in our tDCS study with ADHD patients^49^. Participants had to indicate via button press if the middle target arrow pointed to the right or to the left. The outer arrows served as distractors. The task had about 16 min duration and 528 trials in total with 50% of the trials being congruent and incongruent. Stimuli were presented for 60 ms with an inter stimulus interval of 1676 ms and a trial duration of 1736 ms. Again, prior to the task participants performed a short training at each visit.

Accuracy and RT for both tasks were analysed using the computing environment R^50^ (version 3.6.1). As measurement of accuracy in the 2-back task we computed d′ scores^51^. RT were analysed for target trial hits. For the Flanker task accuracy was defined as proportion of correct responses for congruent and incongruent trials separately. RT as well were analysed for correct responses for congruent and incongruent trials.

### Transcranial direct current stimulation

We applied 2 mA atDCS over the lDLPFC using a Starstim 32 stimulator (Neuroelectrics, Barcelona, Spain). Five 3.14 cm^2^ circular PiStim electrodes were positioned at AF3 (897 µA), AF7 (284 µA), F3 (819 µA), Fp2 (-1000 µA) and T7 (-1000 µA), filled with EEG electrode gel. Electrodes were positioned using a head cap following the 10–10 system (Fig. 2b). For anodal stimulation current was ramped up for 30 s at the beginning and down during 30 s at the end of stimulation, for sham stimulation current was ramped up and immediately down for 60 s at the beginning and end of the stimulation.

#### Montage optimisation

**Figure 3:**
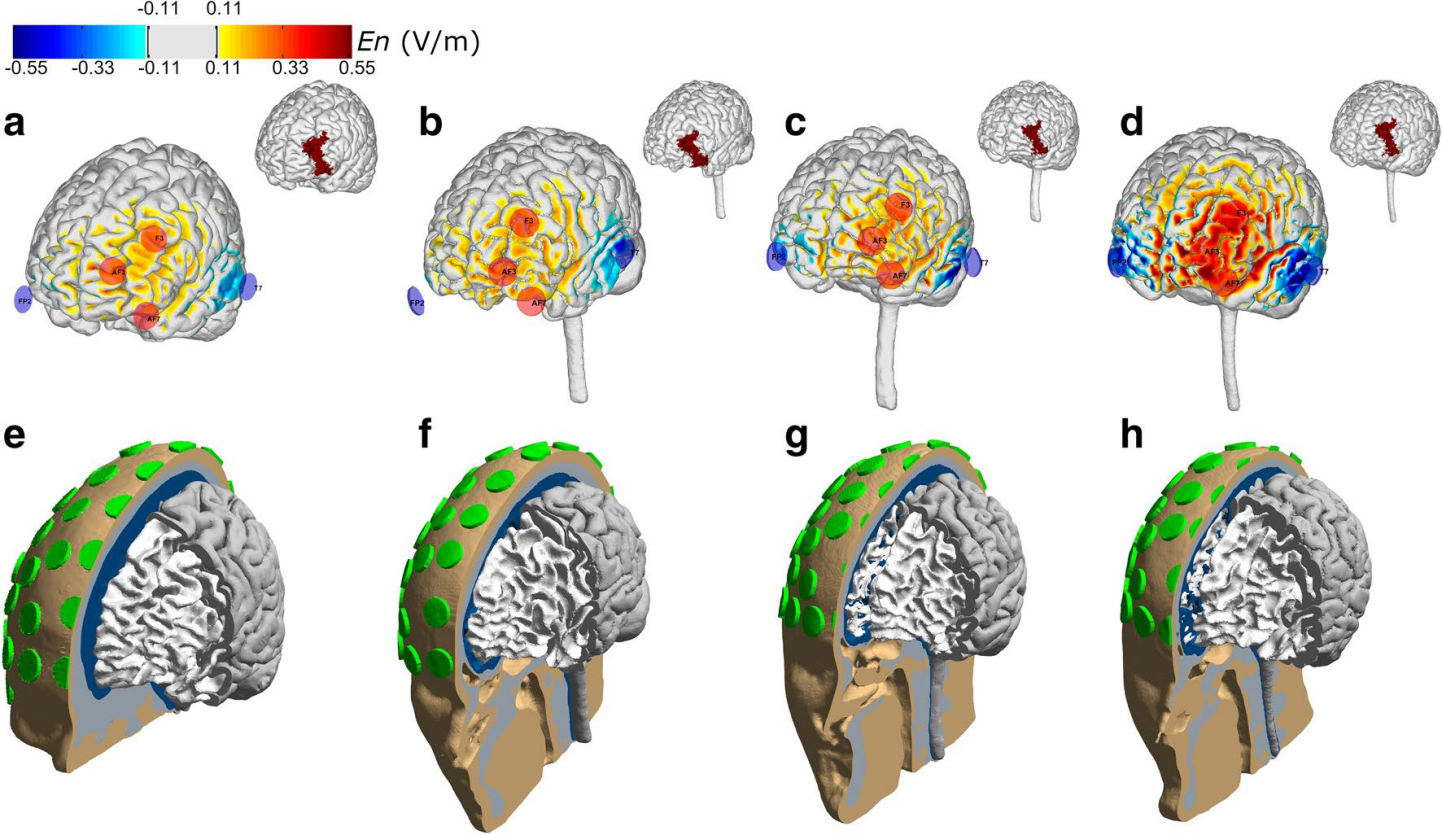
Distribution of the normal component of the E-field in the cortical surface of 4 of the head models used in this study. The distribution induced by the optimised montage on the cortical surface are shown in the top row for: the template head model **(a)** and the participants with the lowest, median and highest average *E*_*n*_ in the left DLPFC ((**b–d**), respectively). The head models for each participant are shown in the bottom row **(e–h)**, including the 61 electrodes present in the headcap used in this study. Figures were created using Comsol (Version v5.3a, https://www.comsol.com/) and MATLAB (Version 2018a, https://www.mathworks.com/).

The multichannel montage used in this study was derived from an optimisation algorithm applied to a template head model (Colin head model)^19^. The optimisation was conducted using the Stimweaver algorithm^52^. This method determines the montage that minimise the least squares difference between a weighted target E-field map and the weighted E-field induced by the montage. In this study, we focused on the component of the E-field normal to the cortical surface (*E*_*n*_) of the head model, since this component is thought to induce the strongest polarisations in the pyramidal neurons, aligned along this direction (lambda-E model)^52^. For the target map we created a maximum excitation region (maximum weight, 10), and a positive target *E*_*n*_ field of + 0.50 V/m on the left hemisphere based on Brodmann area 46/left DLPFC. The target *En*-field on the remaining areas was set to 0 V/m with a lower weight (2). The electrode positions were selected from those available in Neuroelectrics PRO cap (64 positions of the 10–10 EEG system). The maximum current in each electrode was constrained to 1.0 mA and the total injected current was limited to a maximum of 2.0 mA. The distribution of *E*_*n*_ in the cortical surface of the template head model is shown in Fig. 3a.

### EEG recording and preprocessing

EEG was recorded throughout the whole stimulation sessions (T2–T5), i.e. resting state EEG prior to stimulation, EEG during stimulation, resting state EEG after stimulation, EEG during task performance after stimulation (2-back and Flanker task). All EEG data was analysed except for EEG recording during stimulation. EEG was recorded with a rate of 500 S/s and a bandwidth of 0–125 Hz (DC coupled) from 32 channels during the stimulation sessions (see Fig. 2b) using the Starstim 32 stimulator, NG PiStim electrodes and NIC software v2.0.10 and v2.0.11 (Neuroelectrics, Barcelona, Spain). The electrode positions corresponded to the international 10–10 standard system, with the reference and ground electrodes located on the right mastoid. Electrode impedances were kept below 10 kΩ.

We preprocessed EEG data using BrainVisionAnalyzer 2 (Brain Products GmbH, Gilching, Germany). The data was re-referenced to the common average and filtered (120 Hz low-pass, 0.53 Hz high-pass filter, 50 Hz notch filter). We applied a semiautomatic raw data inspection, an independent component analysis and, following segmentation, a semiautomatic artefact rejection to remove artefacts. For the 2-back task a mean number of 191 trials (SD: 35) and for the Flanker task a mean number of 210 trials (SD: 37) was included in the analyses. Further information on trial numbers for both tasks can be found in Tables S2 and S3 in the supplementary.

EEG data was further analysed using the Fieldtrip toolbox^53^. Due to a limited number of target hits in the 2-back task for several participants we restricted our analysis of EEG correlates to correct rejection trials in the 2-back task. However, trial numbers for 2-back target hit trials and results of corresponding ERP analyses can be found in the supplementary. In the 2-back task, we analysed the N2 (200–270 ms post stimulus) in the FCz electrode as region of interest (ROI) and the P3 (250–500 ms post stimulus) component in the Pz electrode as ROI. In the Flanker task, ERPs and time–frequency representations were analysed for correctly answered incongruent trials. Here, we analysed the N2 component (250–350 ms post stimulus) in the FCz electrode and P3 (350–600 ms post stimulus) component in the Pz electrode. ROIs were based on previous investigations of task related ERPs^54–56^. The time windows of all component were defined based on visual inspection of the grand average ERP from all participants.

For task related oscillatory activity we performed a time–frequency analysis with a Hanning taper in steps of 2 Hz for the theta (4–6 Hz), alpha (8–12 Hz) and beta frequency band (14–30 Hz). For all frequency bands event-related synchronization/desynchronization (ERS/ERD)^57^ was computed with respect to a pre stimulus baseline (− 250 to 0 ms). In the 2-back task we analysed the post stimulus interval from 0 to 1000 ms, in the Flanker task we analysed the post stimulus interval from 0 to 700 ms.

Resting state EEG was Fourier-transformed with a moving Hanning window in steps of 0.5 Hz for the theta (4–7.5 Hz), alpha (8–12 Hz) and beta (12.5–30 Hz) frequency band and averaged in every participant. Next, we computed pre to post stimulation changes in frequency power for all frequency bands.

### Calculation of individual E-field distribution

From the included 22 participants 16 individuals underwent structural head scanning on a 3 T Philips Achieva scanner, during which the following sequences were acquired: a T1-weighted scan (1 mm^3^, TR = 2530 ms, TE = 3.5 ms, TI = 1100 ms, FA = 7°, fast water excitation), a T2-weighted scan (1 mm^3^, TR = 3200 ms, TE = 300 ms, no fat suppression), and a diffusion MRI (dMRI) scan (2 mm^3^, TR = 6300 ms, TE = 51 ms, 67 directions, b = 1000). Each participants MRI was segmented using an in-house implementation combining extra-cerebral tissue segmentations from a new segmentation approach, which will be included in a future version of the open-source simulation toolbox SimNIBS^58^, with brain tissue segmentations and cortical gray matter surface reconstructions from FreeSurfer^59^. Finite element head models were then generated, including representations of the scalp, skull, CSF, gray matter and white matter. The models also contained representations of Pistim electrodes (1 cm radius, cylindrical Ag/AgCl electrodes) placed in 61 positions of the 10–10 EEG system. The head models of the template head model and 3 participants are shown in Fig. 3e–h. For the electrodes, only the conductive gel underneath the metal connector was represented in the head model. Unless otherwise stated, the scalp, skull, and CSF were modelled as isotropic with conductivities of 0.33 S/m, 0.008 S/m, and 1.79 S/m, respectively, which are appropriate values for the DC-low frequency values used in transcranial current stimulation^19^. The gray and white matter were modelled as anisotropic (volume normalization)^60^, with isotropic conductivity values used for diffusion tensor scaling of 0.40–0.15 S/m, for the gray matter—white matter^19^. The E-field distribution induced by the common optimised montage was calculated for each participant (see Fig. 3b–d). All E-field calculations were performed in COMSOL, using second- order tetrahedral mesh elements to solve Laplace’s equation. For each participant the surface-average value of the normal component of the E-field was calculated for the lDLPFC (Brodmann area 46), as region targeted by the optimisation. Figure 1b shows the relationship between participants age and the normal component of the E-field.

### Questionnaire on tolerability and participant blinding of atDCS

For assessment of side effects and blinding effectiveness we used a standardised safety questionnaire^61^. Participants rated the incidence and intensity (0 = not experienced to 3 = strongly experienced) of the six most common tDCS side effects on a 4-point scale. Additionally, following each stimulation application, the participants gave their opinion as to whether they had received anodal or sham stimulation.

### Statistical analyses

#### Behavioural data

Throughout all analyses, results were regarded as statistically significant with a two-tailed p value < 0.05. Differences in accuracy and RT were analysed using linear mixed effects models (LME). The models of 2-back task performance during stimulation included the fixed factors *stimulation* (sham, anodal) and *age* and their interaction as well as *visit* (atDCS session 1 to 4). The models of 2-back task performance after stimulation included the additional fixed factor *atDCS* + *target task* (atDCS with or without concurrent 2-back task performance). For the Flanker task we computed two models with the fixed factors *stimulation*, *trial* (congruent or incongruent), *atDCS* + *target task*, *age* and all corresponding interactions, except for the four-way interaction, as well as *visit*. In all models we included a random intercept. In case of violations of assumptions, we computed robust linear mixed models using the robustlmm package^62^.

#### Neurophysiological data

Analyses of neurophysiological data were performed using the Fieldtrip toolbox^53^. Differences in ERPs of correct rejection 2-back trials and of incongruent Flanker trials were investigated using two-way repeated measurement ANOVAs with the factors *stimulation* and *atDCS* + *target task* based on the respective ROIs and time intervals. Regarding task related ERD/ERS and resting state post–pre frequency changes, we used two-way repeated measurement ANOVAs with the factors *stimulation* and *atDCS* + *target task* with a cluster based approach. This non-parametric approach solves the problem of multiple comparisons by cluster correction and avoids assumptions on normally distributed data. In all ANOVAs we restricted our analysis to the main effect of *stimulation* and the interaction of *stimulation* and *atDCS* + *target task*. Due to the number of ANOVAs computed for EEG analysis we used a Bonferroni-Holm correction to adjust the alpha level. Following the ANOVAs, in case of a significant main or interaction effect paired t-tests for the specific significant time window and cluster electrodes or ROI were conducted.

#### Correlation of E-field and stimulation effects

We computed correlations between stimulation effects (difference between the sham and anodal condition) and the individual normal E-field values in the stimulation target area. Due to the high number of correlations a Bonferroni-Holm Alpha correction was performed.

#### Tolerability and blinding of tDCS

Differences in experience of side effects between sham and anodal stimulation were investigated using Wilcoxon signed-rank tests. Blinding effectiveness was assessed using a Pearson’s chi-squared test.

## Results

### Behavioural data

**Figure 4:**
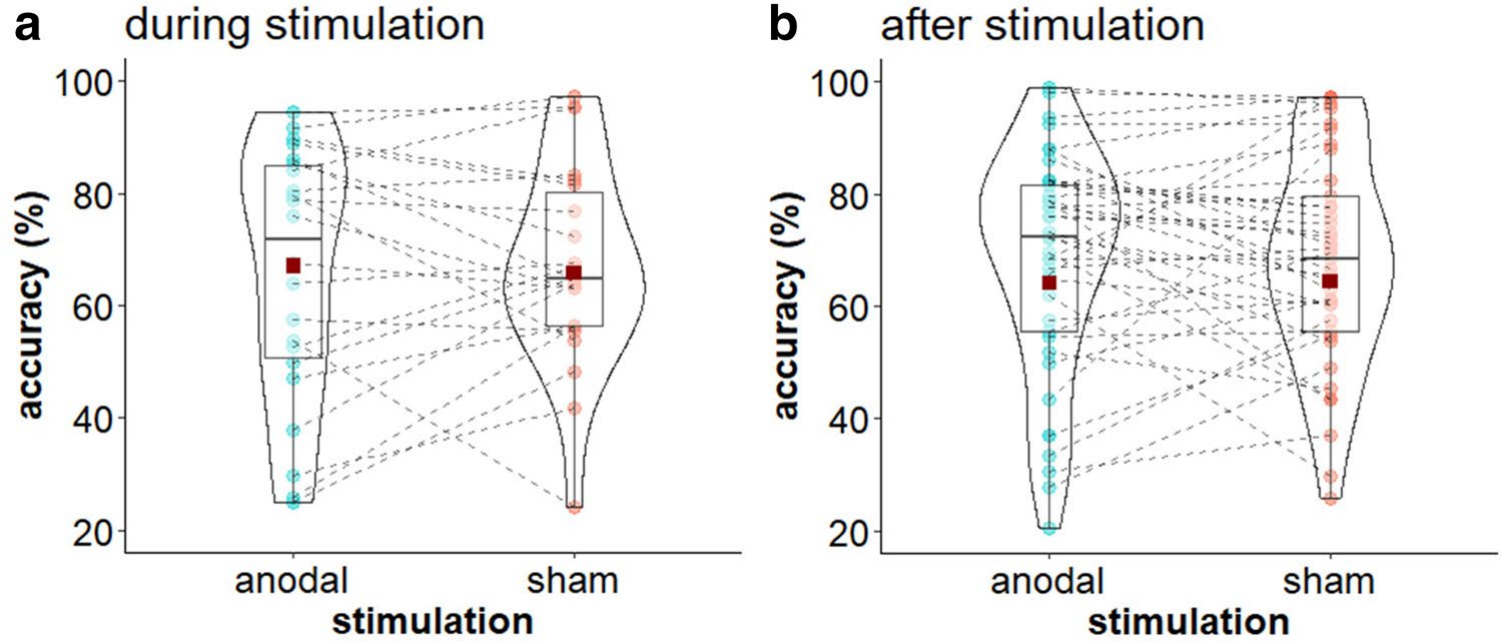
Stimulation effects on 2-back performance. **(a)** Violin plot for mean (± SD) and individual 2-back accuracy for sham and multichannel stimulation during stimulation. **(b)** Violin plot for mean (± SD) and individual 2-back accuracy for sham and multichannel stimulation after stimulation, averaged over concurrent and non-concurrent task condition. Red squares indicate mean accuracy, coloured dots indicate individual accuracy.

Mean accuracy and RT values for both tasks are displayed in Table 2. Additionally, Fig. 4 displays 2-back accuracy during and after stimulation. Results of the LMM of 2-back d′ scores and RT are summarized in Table 3. Neither for 2-back performance during nor after stimulation we found a significant main effect of or interaction with *stimulation* on d′ scores or RT. There was a significant effect of *age* for d′ scores during stimulation, showing an increase in d′ scores with increasing age (β = 0.162, *t* (19.8) = 2.22, *p* = 0.038). For 2-back RT after stimulation we found a reduction of RT at the third stimulation visit compared to the overall RT mean (β =  − 24.386, *t* (57) =  − 2.55, *p* = 0.013).

**Table 2:** Mean (Standard deviation) for 2-back d′ score, accuracy (%) and reaction times (ms) and Flanker accuracy (%) and reaction times (ms).

Task	Time point	Outcome	Stimulation condition	
			sham	anodal
2-back	During	d′	2.38 (0.78)	2.35 (0.71)
		Accuracy	67.02 (18.01)	65.75 (22.64)
		RT	580.21 (155.71)	577.43 (173.37)
	After	d′	2.23 (0.78)	2.21 (0.81)
		Accuracy	64.18 (22.94)	64.37 (23.57)
		RT	594.12 (197.29)	566.71 (189.41)
Flanker	After	Accuracy	92.83 (11.35)	94.07 (6.66)
		RT	521.43 (129.44)	512.08 (126.82)

*RT* reaction time.

**Table 3:** Coefficients and corresponding t values and p values for 2-back accuracy (d′) and reaction time during and after stimulation.

Predictors	Accuracy			Reaction time		
	β	t value	p value	β	t value	p value
**During stimulation**						
Stimulation (sham vs. anodal)	− 0.016	− 0.258	0.799	− 2.068	− 0.299	0.769
Age	**0.162**	**2.223**	**0.038**	22.041	1.256	0.224
Visit 1	− 0.172	− 1.234	0.231	− 3.995	− 0.244	0.810
Visit 2	0.025	0.202	0.842	− 16.325	− 1.140	0.269
Visit 3	0.019	0.152	0.880	26.069	1.775	0.093
Stimulation × age	− 0.001	− 0.023	0.982	− 0.144	− 0.038	0.970
**After stimulation**						
Stimulation (sham vs. anodal)	− 0.016	− 0.485	0.634	− 8.478	− 1.545	0.128
Task (con vs. non-con)	0.049	1.453	0.162	11.058	2.005	0.050
Age	0.033	0.720	0.479	− 4.857	− 0.632	0.529
Visit 1	0.000	0.003	0.997	19.157	1.914	0.061
Visit 2	0.088	1.492	0.151	0.693	0.073	0.942
Visit 3	0.039	0.673	0.510	− **24.386**	− **2.553**	**0.013**
Stimulation × task	0.007	0.215	0.832	− 4.193	− 0.760	0.450
Stimulation × age	− 0.034	− 1.825	0.086	− 2.307	− 0.757	0.453
Concurrent × age	0.009	0.448	0.659	− 0.505	− 0.166	0.869
Stimulation × task × age	0.004	0.233	0.818	− 1.580	− 0.510	0.612

Significant results are printed in bold.

**Figure 5:**
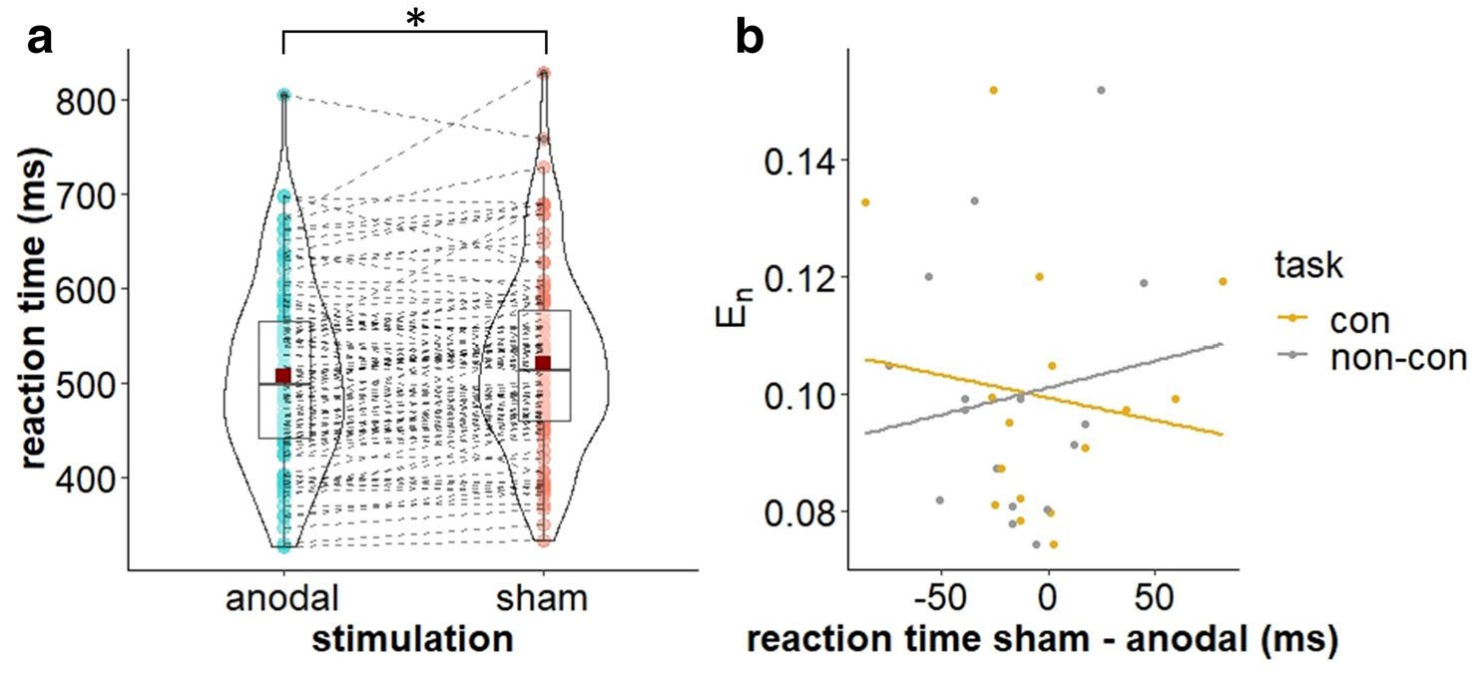
Stimulation effects on Flanker performance. **(a)** Violin plot for mean (± SD) and individual Flanker RT for sham and multichannel stimulation, averaged over concurrent and non-concurrent task condition. Red squares indicate mean RT, coloured dots indicate individual RT (*p < 0.05). **(b)** Scatter plots with regression lines of stimulation effect (sham-anodal) on Flanker RT and individual normal E-field component (E_*n*_) for concurrent (con) and non-concurrent (non-con) task condition.

Analysis of the Flanker task showed no *stimulation* effect on accuracy but an effect of *stimulation* on RT (β =  − 6.438, *t* (57) =  − 2.47, *p* = 0.015; see Table 4). Following anodal stimulation, RT were generally reduced compared to sham stimulation (see Fig. 5a). Importantly, there was no trade-off between RT and accuracy, meaning while RT were reduced following anodal compared to sham stimulation, accuracy did not decrease. Furthermore, we found an interference effect reflected in a significant effect of *trial* for both accuracy (β =  − 2.145, *t* (138) =  − 10.42, *p* < 0.001) and RT (β = 35.701, *t* (138) = 13.81, *p* < 0.001): Compared to congruent trials accuracy scores were lower and RT were higher for incongruent trials. Besides, we found a significant interaction of *trial* and *age* for RT (β = 3.298, *t* (138) = 2.36, *p* = 0.019). While RT decreased with increasing age for both trial types, this decrease was steeper for congruent than for incongruent trials.

**Table 4:** Coefficients and corresponding t values and p values for Flanker accuracy and reaction time.

Predictors	Accuracy			Reaction time		
	β	t value	p value	β	t value	p value
Stimulation (sham vs. anodal)	0.103	0.498	0.619	− **6.438**	− **2.472**	**0.015**
Task (con vs. non-con)	− 0.129	− 0.622	0.535	− 0.428	− 0.163	0.870
Trial (congr. vs. incongr.)	− **2.145**	− **10.420**	** < 0.001**	**35.700**	**13.804**	** < 0.001**
Age	0.168	0.720	0.473	3.601	0.987	0.325
Visit 1	− 0.546	− 1.460	0.147	8.616	1.813	0.072
Visit 2	− 0.275	− 0.766	0.445	7.161	1.583	0.116
Visit 3	0.354	0.982	0.328	− 6.078	− 1.340	0.182
Stimulation × task	− 0.004	− 0.021	0.983	0.738	0.282	0.778
Stimulation × trial	− 0.025	− 0.123	0.902	0.185	0.072	0.943
Concurrent × trial	− 0.298	− 1.447	0.150	0.110	0.043	0.966
Stimulation × age	− 0.225	− 1.961	0.052	− 0.328	− 0.226	0.821
Task × age	0.185	1.607	0.110	1.864	1.287	0.200
Trial × age	0.057	0.511	0.610	**3.298**	**2.364**	**0.019**
Stimulation × task × trial	− 0.295	− 1.432	0.154	1.479	0.571	0.569
Stimulation × task × age	− 0.014	− 0.119	0.905	− 2.092	− 1.421	0.157
Stimulation × trial × age	0.004	0.032	0.975	0.085	0.061	0.951
Task × trial × age	0.114	1.023	0.308	− 1.175	− 0.842	0.401

Significant results are printed in bold.

### Neurophysiological data

The ANOVAs of 2-back and Flanker ERPs did not show a significant effect of *stimulation* or a significant stimulation*atDCS + *target task* interaction.

**Figure 6:**
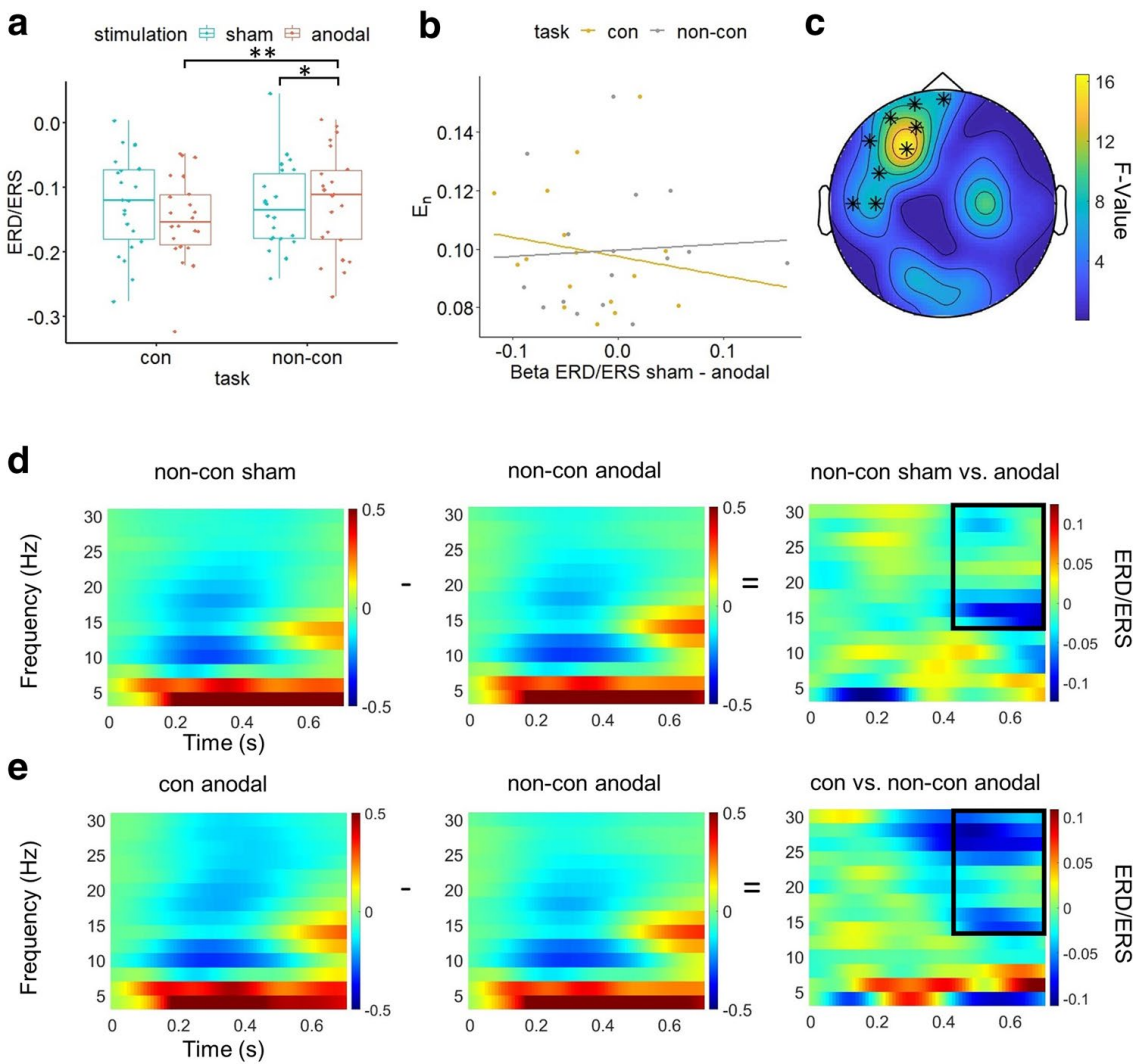
Stimulation*atDCS + target task interaction effect on Flanker task related beta ERD/ERS. **(a)** Boxplots of individual beta ERD/ERS averaged over channels and time in significant stimulation*atDCS + *target task* interaction cluster (AF7, Fp1, AF3, C5, Fpz, F3, F7, FC5, T7; 430–700 ms) for sham and anodal stimulation, separated for concurrent (con) and non-concurrent (non-con) task condition (*p < 0.05, **p < 0.01). **(b)** Scatter plot with regression lines of stimulation effect (sham-anodal) on Beta ERD/ERS and individual normal E-field component (E_n_) for concurrent (con) and non-concurrent (non-con) task condition. **(c)** Topography of significant stimulation*atDCS + *target task* interaction effect of Flanker related beta ERD/ERS marked by asterisks. **(d)** Flanker task related time–frequency representation (TFR), averaged across electrodes forming significant stimulation*atDCS + *target task* interaction cluster. From left to right: Flanker task TFR following non-concurrent sham stimulation, TFR following non-concurrent anodal stimulation, difference in TFR for non-concurrent condition between sham and anodal stimulation. The black box indicates the significant difference in beta oscillatory power between both conditions. **(e)** Flanker TFR, averaged across electrodes forming significant stimulation*atDCS + *target task* interaction cluster. From left to right: Flanker task TFR following concurrent anodal stimulation, TFR following non-concurrent anodal stimulation, difference in TFR for anodal stimulation condition between concurrent and non-concurrent task condition. The black box indicates significant difference in beta oscillatory power between both conditions. Figures were created using MATLAB (Version 2018a, https://www.mathworks.com/), FieldTrip (Version 20190712, https://www.fieldtriptoolbox.org/) and the computing environment R (Version 3.6.1, R Core Team (2019), https://www.R-project.org/).

Similarly, our analyses of 2-back task related alpha, theta and beta ERD/ERS revealed no significant *stimulation* effect or stimulation*atDCS + *target task* interaction. For the Flanker task ERD/ERS our analyses did not show a main effect of *stimulation* but a significant interaction effect of stimulation*atDCS + *target task* for beta ERD/ERS in an area covering the stimulation target area (AF7, Fp1, AF3, C5, Fpz, F3, F7, FC5, T7; 430–700 ms; see Fig. 6c). Pairwise comparisons showed increased oscillatory beta power following anodal compared to sham stimulation in the non-concurrent atDCS + target task condition (t =  − 2.744, p = 0.014; see Fig. 6a,d,e). Additionally, following anodal stimulation beta power was increased for non-concurrent compared to the concurrent tDCS + target task condition (t =  − 4.16, p = 0.002).

Two-way repeated measures ANOVAs of resting state frequency data did not show a significant *stimulation* effect for any frequency band. Also, the interaction of stimulation*atDCS + *target task* was not significant in all frequency bands.

### Correlation of E-field and stimulation effects

We did not find a significant correlation between the individual normal component of the E-field and stimulation effects on behavioural or neurophysiological outcomes. Especially the significant stimulation effect on Flanker RT was not correlated with the individual E-field component (see Fig. 5b), neither for the concurrent stimulation condition (*r* =  − 0.13, *p* = 0.612) nor for the non-concurrent *atDCS* + *target task* condition (*r* = 0.13, *p* = 0.619). The same was true for the stimulation effect on Flanker task related beta oscillatory power and the individual E-field component for the concurrent (*r* =  − 0.15, *p* = 0.578) and non-concurrent *atDCS* + *target task* condition (*r* = 0.06, *p* = 0.816; see Fig. 6b).

Additionally, participants age and the individual E-field component were not correlated (see Fig. 1b).

### Tolerability and blinding of tDCS

For the conditions sham tDCS without concurrent task (χ^2^ (1, N = 22) = 0.18, p = 0.669), sham tDCS with concurrent task (χ^2^ (1, N = 22) = 1.81, p = 0.179) and atDCS with concurrent task (χ^2^ (1, N = 20) = 2.91, p = 0.088) participants were unable to guess better than chance whether they had received anodal or sham stimulation, while for the atDCS without concurrent task condition the rate of correct assumptions was significantly higher than guess probability (χ^2^ (1, N = 22) = 4.54, p = 0.033). The intensity of perceived side effects was generally low (see Supplemental Table S1). Only the intensity of perceived itching during stimulation was significantly higher under anodal compared to sham stimulation (*z* = 2.52, *p* = 0.012).

## Discussion

The goal of the present study was to examine the effects of multichannel atDCS targeting the lDLPFC in children and adolescents, investigating the influence of concurrent task performance during stimulation and individual anatomy. We could show that (1) atDCS did not affect WM performance in a 2-back task (target task). Additionally, atDCS did not affect neurophysiological activity in this 2-back task (note, analyses for 2-back neurophysiological activity were based on correct rejection trials since there were not enough trials to calculate ERP components for hit targets). (2) In a Flanker task (non-target task) we found a reduction of RT following atDCS. (3) This effect was independent of whether atDCS had been combined with a cognitive task (2-back task). However, increased Flanker task related beta oscillation was observed only following stimulation without concurrent task performance. (4) The individual E-field was not correlated with stimulation effects. (5) The stimulation led to minor side effects.

Our partly null finding could be due to a suboptimal combination of stimulation parameters. Based on computational modelling, a multichannel montage leads to a more focused stimulation while a classical bipolar montage leads to a comparatively diffuse current flow in the brain^20,22^. However, it is not clear whether a more focal stimulation also causes stronger tDCS effects. WM is a cognitive process based on a widely distributed neural network^63,64^. A multichannel montage may not be able to sufficiently activate this underlying network due to its focality, which may lead to reduced effects on performance, while larger electrodes allow simultaneous activation of different relevant brain regions^65^.

Furthermore, the 2 mA total injected current used in this study may not have been sufficient in every participant in terms of induced E-field strength in the target area to lead to detectable tDCS effects^66^. More important than simply increasing the current intensity seems to be the individualisation of the stimulation in order to ensure that a sufficient and at the same time safe current intensity is applied to each participant^22,67^. The multi-channel montage we used was optimised for the target region lDLPFC, but only based on a standard brain. A next step would be to individualise the optimisation with respect to the participant specific neuroanatomy.

Regarding WM performance, imaging studies prove that the activation of the DLPFC is dependent on cognitive load^68^, with higher cognitive load leading to stronger neural activation and potentially stronger tDCS effects. However, the DLPFC activation is related to the individual WM capacity^69^ and shows a nonmonotonic, inverted-U response to WM load^70^. Therefore, an excessive cognitive load could also be an inhibitor for a possible tDCS effect^34^. Compared to a standard 2-back task, the task we used was complicated by the requirement to respond to each stimulus, not only target stimuli, and the use of lures. The 2-back accuracy during stimulation was shown to be dependent on the age of participants, which may indicate a possible overload in younger children. Thus, it can be hypothesised that some participants were overchallenged by the task and the optimum of their DLPFC activation had already been exceeded.

In terms of neurophysiological activity related to WM performance, we cannot draw definite conclusions since we were not able to analyse target hit trials in the 2-back task. Here, EEG analyses were based on correct rejection trials that do not represent the primary measure of n-back tasks.

Results of the resting-state EEG recordings showed that theta, alpha and beta power did not differ significantly between anodal and sham tDCS conditions. These results correspond to previous studies in adults demonstrating no atDCS effect on resting state neurophysiological activity^36–38^. The missing atDCS influence on resting state EEG might be due to a state dependency of stimulation effects^71,72^. Besides, resting state EEG was recorded directly after termination of stimulation. This might have been too early for long term tDCS after-effects to occur. Future studies should include additional resting state EEG recordings following task performances after stimulation. In this way, long-term effects could be revealed, as they have already been found in previous studies^33,73,74^.

In contrast to the 2-back task, we found an atDCS effect on behavioural and neurophysiological outcomes in the non-target Flanker task. Our results are consistent with previous studies in adults that also found reduced RT in a Flanker task after atDCS over the lDLPFC^32,75^. Still, it should be emphasised that the stimulation effects in the Flanker task were small and limited to RT and specific neuronal oscillations. As for WM functioning, the functions relevant for the Flanker task, such as interference control, sustained attention and response inhibition, are based on a network of different brain areas. This network includes the DLPFC and the anterior cingulate cortex as critical hubs^76^. We cannot exclude the possibility that our multichannel montage activated the circuits involved in the Flanker task more strongly than the 2-back related WM network due to the distribution of the electrodes on the skull.

Interestingly, the atDCS effect on Flanker task performance occurred regardless of whether atDCS had been combined with a cognitive task or not. The fact that improvements in Flanker task performance were also found after atDCS with concurrent 2-back task performance argues against task-specific effects of stimulation, as assumed by the activity selectivity model^77,78^. The Flanker task is mainly applied to investigate interference control, but also relies on WM processes. Working memory, in turn, is composed of different executive functions^79,80^. While the 2-back task particularly requires an updating component^81^, the Flanker task especially requires an inhibition component^82^. Both WM components seem to be closely connected functionally^83,84^ and structurally^85^, which is why a transfer of stimulation effects is conceivable. Additionally, following the flexible hub theory, a general enhancement of the lDLPFC activity could contribute to the improvement in different tasks^30,31^. At the same time, the changes in Flanker task performance cannot be clearly attributed to stimulation in isolation since this task was performed as second task after the application of stimulation and 2-back task performance. This increased strain on the target area lDLPFC through the stimulation and the following execution of the 2-back task after stimulation might have had effects on the neuroplasticity and thus on the performance in the Flanker task^86^.

Besides the behavioural changes, we found increased Flanker task related beta activity. In the prefrontal cortex a connection of beta oscillations with WM, interference control and distraction prevention has been observed before^87–90^. Studies in adults suggest an activity selectivity of neurophysiological changes following tDCS combined with task performance^38,91^. In our study, the 2-back task during stimulation could also have led to more selective activation, which may have counteracted a transfer effect. However, in this case, neurophysiological activity seems to be more sensitive to this influence than behavioural activity. Therefore, regarding our hypothesis that the effects of stimulation depend on the performance of a task during stimulation, we cannot draw a definite conclusion.

We did not find a connection between atDCS effects and calculations of the individual E-field. This could be because we computed the individual E-field only for a subsample, which may have masked possible correlations. Besides, we determined the participant-specific stimulation effect only in relation to the sham condition. Other studies that have investigated the relationship between individual E-field and tDCS effects have mostly determined the stimulation effect in relation to a pre-stimulation baseline^17,92^. In this way, tDCS effects can be examined more precisely, for example by considering the participants day-specific individual performance level.

In children and adolescents, a 2 mA multichannel montage has not been investigated so far. In the current study, all participants reported only mild side effects directly following stimulation. Nevertheless, our study demonstrates the importance to adapt atDCS for applications in a paediatric group, also regarding safety aspects. The case of a female participant, who developed an epileptic disease during her participation in this study, shows that the screening procedure needs to ask more specifically for neurological abnormalities^40,93^. Additionally, our study demonstrates the importance to proactively ask for adverse events following stimulation sessions, as it is procedure in clinical trials.

There are some limitations in our study. Due to a relatively large drop-out, we were only able to include a comparatively small sample (n = 22) in the evaluation. Furthermore, the E-field distribution was only computed for a subsample. In particular, the lack of correlation between individual E-field distributions and atDCS effects can therefore only be interpreted to a limited extent. To draw stronger conclusions regarding this relationship in children and adolescents, future studies should examine a larger sample size. At the same time, experiments on children should start with small samples to avoid exposing a large population to potential risks. Furthermore, the acquisition of a task baseline at each visit would have been an additional control for intraindividual differences. Especially the investigation of the influence of individual E-field on atDCS effects would have benefited from such a baseline. Still, we were able to determine stimulation effects with regards to sham stimulation. Another limitation is that participants were not effectively blinded whether they had received anodal or sham stimulation in the non-concurrent atDCS condition. We used a control task to differentiate stimulation effects more clearly. However, using an additional active stimulation control condition (e.g. cathodal tDCS) would have been beneficial. In this way, stimulation effects could have been more clearly delineated and effects attributable to lack of blinding more clearly excluded. However, the atDCS effect on Flanker task performance was independent of whether atDCS was applied with concurrent or non-concurrent task performance. Besides, no stimulation effect in the target 2-back task was observed.

Our study demonstrates limited but at the same time transfer effects of multichannel atDCS targeting the lDLPFC in children and adolescents. While our behavioural data argues against an activity selectivity effect of stimulation, neurophysiological activity might be more sensitive regarding concurrent task performance than behavioural outcomes. Further, tDCS studies in children and adolescents should use screening procedures adapted to this age group and inquire about adverse events after each stimulation session, to prevent dangers from the stimulation.

## Supplementary Information

Supplementary Table S1.

Supplementary Table S2.

Supplementary Table S3.

Supplementary Table S4.
