# Supplementary material for: Multichannel anodal tDCS over the left dorsolateral prefrontal cortex in a paediatric population
Source: Sci Rep. 2021 Nov 2;11:21512. doi: 10.1038/s41598-021-00933-z (PMC8563927; doi:10.1038/s41598-021-00933-z)
Supplement: Supplementary file 1 — Supplementary Table S1. [file 41598_2021_933_MOESM1_ESM.docx]

Table S1

*Mean intensity (scale 0–3, Ø) and comparison of side effects for anodal and sham stimulation.*

|  | sham | anodal | z | p |
| --- | --- | --- | --- | --- |
| Itching sensation | **0.38** | **0.81** | **- 2.51** | **0.012** |
| Pain | 0.23 | 0.35 | - 1.01 | 0.314 |
| Burning sensation | 0.23 | 0.42 | - 1.41 | 0.161 |
| Warmth/Heat | 0.04 | 0.19 | - 1.62 | 0.105 |
| Metallic/Iron taste | 0.02 | 0.00 | 0 | 1 |
| Fatigue/Decreased alertness | 0.97 | 0.92 | 0.09 | 0.923 |
