# Supplementary material for: Multichannel anodal tDCS over the left dorsolateral prefrontal cortex in a paediatric population
Source: Sci Rep. 2021 Nov 2;11:21512. doi: 10.1038/s41598-021-00933-z (PMC8563927; doi:10.1038/s41598-021-00933-z)
Supplement: Supplementary file 2 — Supplementary Table S2. [file 41598_2021_933_MOESM2_ESM.docx]

Table S2

*Mean number, Standard-Deviation (SD) and Minimum (Min) and Maximum (Max) for 2-back task correct rejection trials included in EEG analyses for all four stimulation conditions.*

|  | Nonconcurrent Sham | Concurrent Sham | Nonconcurrent Verum | Concurrent Verum |
| --- | --- | --- | --- | --- |
| Mean | 186.5 | 192.3 | 194.2 | 193.7 |
| SD | 45.9 | 30.6 | 30.6 | 32.2 |
| Min | 50 | 118 | 118 | 125 |
| Max | 238 | 240 | 235 | 251 |
