# Supplementary material for: Multichannel anodal tDCS over the left dorsolateral prefrontal cortex in a paediatric population
Source: Sci Rep. 2021 Nov 2;11:21512. doi: 10.1038/s41598-021-00933-z (PMC8563927; doi:10.1038/s41598-021-00933-z)
Supplement: Supplementary file 3 — Supplementary Table S3. [file 41598_2021_933_MOESM3_ESM.docx]

Table S3

*Mean number, Standard-Deviation (SD) and Minimum (Min) and Maximum (Max) for Flanker task for correctly answered incongruent trials included in EEG analyses for all four stimulation conditions.*

|  | Nonconcurrent Sham | Concurrent Sham | Nonconcurrent Verum | Concurrent Verum |
| --- | --- | --- | --- | --- |
| Mean | 207.9 | 209.2 | 205.6 | 220.1 |
| SD | 43.4 | 43.3 | 33.4 | 23.9 |
| Min | 102 | 78 | 127 | 171 |
| Max | 257 | 253 | 247 | 266 |
