# Supplementary material for: Multichannel anodal tDCS over the left dorsolateral prefrontal cortex in a paediatric population
Source: Sci Rep. 2021 Nov 2;11:21512. doi: 10.1038/s41598-021-00933-z (PMC8563927; doi:10.1038/s41598-021-00933-z)
Supplement: Supplementary file 4 — Supplementary Table S4. [file 41598_2021_933_MOESM4_ESM.docx]

**Supplementary ERP Analyses**

The ANOVAs of 2-back target hit trial ERPs did not show a significant effect of *stimulation* or a significant *stimulation*atDCS+target task* interaction.

Table S4

*Number of 2-back target hit trials per participant and stimulation condition.*

| Subject | Nonconcurrent Sham | Nonconcurrent Sham | Nonconcurrent Verum | Nonconcurrent Verum |
| --- | --- | --- | --- | --- |
| 001fK | 31 | 46 | 59 | 14 |
| 002mK | 99 | 96 | 91 | 103 |
| 003fK | 74 | 102 | 78 | 91 |
| 004mK | 42 | 40 | 24 | 27 |
| 005fK | 102 | 98 | 104 | 96 |
| 005mK | 36 | 48 | 52 | 32 |
| 007mK | 60 | 74 | 65 | 67 |
| 009mK | 55 | 45 | 59 | 64 |
| 010fK | 78 | 56 | 40 | 87 |
| 011fK | 67 | 41 | 20 | 67 |
| 013fK | 26 | 50 | 50 | 27 |
| 014mK | 62 | 67 | 80 | 84 |
| 015fK | 71 | 73 | 80 | 71 |
| 015mK | 35 | 21 | 49 | 14 |
| 017fK | 6 | 14 | 21 | 29 |
| 019fK | 88 | 48 | 72 | 65 |
| 023mK | 56 | 57 | 66 | 72 |
| 025fK | 24 | 15 | 16 | 13 |
| 028fK | 68 | 49 | 77 | 59 |
| 029fK | 79 | 70 | 79 | 55 |
| 031fK | 96 | 85 | 63 | 56 |
| 035fK | 39 | 44 | 69 | 71 |
